# Surface Modification of Polyester Fabrics by Ozone and Its Effect on Coloration Using Disperse Dyes

**DOI:** 10.3390/ma14133492

**Published:** 2021-06-23

**Authors:** Rafaela Stefanie Gabardo, Dayane Samara de Carvalho Cotre, Manuel José Lis Arias, Murilo Pereira Moisés, Bruna Thaisa Martins Ferreira, Rafael Block Samulewski, Juan P. Hinestroza, Fabricio Maestá Bezerra

**Affiliations:** 1 Textile Engineering (COENT), Universidade Tecnológica Federal do Paraná (UTFPR), Apucarana 86812-460, Brazil; rafaelagabardo@alunos.utfpr.edu.br (R.S.G.); dayanecarvalho@utfpr.edu.br (D.S.d.C.C.); fabriciom@utfpr.edu.br (F.M.B.); 2 Textile Research Institute of Terrassa (INTEXTER-UPC), Terrassa, 0822 Barcelona, Spain; 3 Chemistry Graduation (COLIQ), Universidade Tecnológica Federal do Paraná (UTFPR), Apucarana 86812-460, Brazil; murilomoises@utfpr.edu.br (M.P.M.); samulewski@utfpr.edu.br (R.B.S.); 4 Graduate Program in Chemical Engineering (PEQ-UEM), State University of Maringá (UEM), Maringá 87020-900, Brazil; brunathaisaeng@gmail.com; 5 Department of Fiber Science and Apparel Design, Cornell University, Ithaca, NY 14853, USA; jh433@cornell.edu

**Keywords:** dyeing, surface modification, ozone, polyester

## Abstract

Polyester fibers (PES) are the most consumed textile fibers due to their low water absorption; non-ionic character and high crystallinity. However, due to their chemical structure, the chemical interactions between polyester, finishing products, and dyes are quite challenging. We report on the use of ozone to modify the surface of polyester fibers with the goal of improving the interaction of the modified surface with finishing compounds and dyes. We used C.I. Disperse Yellow 211 to dye ozone-treated polyester fabrics and evaluated the effects of ozone treatment using FTIR-ATR, Raman spectroscopy, SEM imaging, rubbing tests, and capillarity measurements. We evaluated the dyeing performance via color analysis, and determined the dyeing kinetics. Experimental results indicate that the modification of polyester fabrics with ozone is a feasible pre-treatment that improves dyeing efficiency allowing better solidity of color and a decrease in the amount of dye required.

## 1. Introduction

Most waste generated by the textile industry [1], is composed of organic and inorganic compounds generated during textile dyeing [2,3]. These compounds are difficult to eliminate by traditional effluent treatment processes [2,4,5,6], due to the excessive content of suspended solids, and the presence of surfactants, detergents, and dyes [7]. The textile sector is also characterized by high competition, stimulated by economic, consumerism, and globalization factors, seeking improvements to reduce its expenses and costs, maintaining the quality of the product and contributing to sustainability [8].

Among the materials used by the textile industry, polyester is considered the most consumed synthetic fiber in the world, being able to integrate different types of products that unfold in the textile chain [9]. However, the dyeing of polyester fibers with dispersed dyes is a complex process as it resembles a solid-on-solid dispersion and encounters considerable difficulties due to the absence of reactive sites, which gives this type of fiber a hydrophobic character [10]. In this context, it is possible to make a modification on the polyester fiber in order to improve its hydrophilicity and, consequently, facilitate the absorption of the dyes.

Many publications have shown the difficulty for treatment of textile effluents, pointing out to the excess content of solids present, in addition of surfactants, detergents, and dyes [3,6,9,10]. An alternative to facilitate treatment is to reduce the consumption of dyes without losing the characteristics of the article, especially the coloring. Thus, surface modification processes can create reactive sites and increase the adsorption of dye by the fiber hence reducing the amount of pollutants in the effluents. In the search for new technologies to improve the dyeing process, the use of ozone gas (O_3_) has emerged as a viable alternative [11], as ozone treatments have the capability to improve the wettability of the polyester fibers [12,13], and to use less water during the dyeing process [9,14,15,16,17].

According to Wakida et al. [18], ozone treatments alter both, the fiber’s surface and its internal structure [19]. Several authors [10,17,20] have also found that ozone treatment has the potential of lower energy consumption during the processing of polyester fibers.

Thus, it is expected that from the use of polyester surface modification with ozone, it will be possible to obtain a more hydrophilic fabric, making it possible to have a dyeing process with less amounts of dye. The absorption of dyes into porous media comprises several steps. Initially, there is mass transfer from the bulk liquid media (dyeing bath), through the boundary layer to the external surface of the fiber, followed by diffusional dye transport inside the fiber.

Kinetic models describing this absorption phenomena use two approaches: (i) Models based on adsorption reaction models such as the pseudo-first order (PFO) [21] and pseudo second order (PSO) [22], models which assume that the adsorption process is exclusively controlled by the adsorption rate of the solute on the surface of the adsorbent, and neglect intraparticle diffusion and external mass transfer; (ii) intra-particle diffusion models, which assume that equilibrium between fluid and surface concentrations of dye, are instantaneously reached inside the pores [23], therefore simplifying the dyeing process to a simple mass transfer process.

In this paper, we report on the use of ozone gas to modify surface of polyester fibers, and the effect of this ozone pre-treatment on the dyeing of these fibers using disperse dyes.

## 2. Materials and Methods

### 2.1. Materials

Polyester plain weave 100% (160 ± 5 gm^−2^), 20 Tex warp yarn (24 yarns/cm), 6.25 Tex weft yarn (24 yarns/cm) were provided by the Polytechnic University of Catalunya (UPC), C.I. Disperse Yellow 211 dye was provided by Golden Technology (São José dos Campos, Brazil). Blue Turquoise Solae GLL, (NH_4_)_2_SO_4_, and sodium bisulphite, were purchased from Sigma Aldrich (Saint Louis, MO, USA).

### 2.2. Polyester Modification by O_3_

Ozone modification was performed using a UV-SURF X4 (UV-Consulting Peschl España, Spain) equipment, 17 W power, and an emission spectrum varying from 185 to 254 nm. Polyester samples of 10 cm × 20 cm were inserted into the equipment’s chamber and exposed for 20, 30, and 45 min to ozone produced by low-pressure mercury lamps.

### 2.3. Evaluation of Modified Fabric

FTIR-ATR spectroscopy was performed using a Frontier—Perkin Elmer, 64 scans with a resolution of 1 cm^−1^ using attenuated total reflectance (ATR) in the range between 650 and 4000 cm^−1^. Raman spectroscopy measurements were carried out using an Alpha 300 R spectrometer—Witec, containing a double monochromator, a 532 nm laser and a microscope with a 20× objective lens; 532 nm laser excitation lines were used. The laser power on the surface of the samples was approximately 7.5 mW cm^−2^ with an integration time of 3 s and a total of 10 scans. Zeta potential measurements were obtained in a Zetasizer Nano from Malvern Instruments. The readings were performed in triplicate on both sides of each sample at pH 6 and 25 °C. For the capillarity tests, a method was adapted from standard JIS L 1907—(Testing methods for water absorbency of textiles). Samples were cut into 20 × 2.5 cm strips and 1 cm of this strip was immersed in a solution containing a reactive dye Blue Turquoise Solae GLL. After 10 min, the height of the dye absorbed by capillarity was measured.

### 2.4. Dyeing

We perform the exhaustion dyeing experiments in a Mathis ALT-1-B mug machine, with a bath ratio of 1:30 (m:v) and 5 g of fabric sample. Total of 2 g·L^−1^ of ammonium sulfate (pH control 5–5.5), 1.5 g·L^−1^ of non-ionic wetting agent, and 1% of w (over fiber weight) of C.I. Dispersed yellow 211 dye were added at 130 °C for 30 min. At the end of the process, reductive washing was performed using a solution containing 2 g·L^−1^ of sodium hydrosulfite and 3 g·L^−1^ of sodium hydroxide (50° BÉ) at 80 °C for 20 min.

### 2.5. Evaluation of Polyester Fabrics after Dyeing

#### 2.5.1. Color Rating

The color evaluation of the samples was performed using a DataColor spectrophotometer, spectraflesh model SF650X, and the i7 Delta Color software. The evaluation was performed under illuminant D_65_, which generates trichromatic coordinates, arranged in the CIE L*a*b* space. The color difference between the treated and untreated samples was calculated using ΔE and ΔE_cmc_.

The color strength was calculated using Equation (1) and the percentage of dye reduction was obtained via Equation (2).

(1) $$Strenght\left(\%\right)=\left(\;\frac{\frac{K}{S}\;sample}{\frac{K}{S}\;standard}\right)\times 100$$

where *K*/*S* is assumed to represent the color intensity.

(2) $$Q\%=\frac{100-Strenght\;\left(\%\right)}{Strength\;\left(\%\right)}$$

For Equation (2), in order to obtain the same color strength, if *Q*% > 0, it indicates that the amount of dye required must be increased and for values of *Q*% < 0, the amount of dye must be reduced.

#### 2.5.2. Morphological Analysis

The surface of the fabric samples was imaged using a scanning electron microscopy (SEM Quanta 250) with an accelerating voltage of 20 kV, Spot 3.5, and magnification levels of 1500 and 4000×.

#### 2.5.3. Fastness to Rubbing Tests

The color fastness to rubbing test was carried out on a KIMAK crock meter (Kimak, Brazil), under dry and wet conditions. The specimens were compared to the gray scale (BSI Standards) in accordance to the Test for colour fastness—Part X12: Colour fastness to rubbing, ISO 105-X12 [24].

#### 2.5.4. Dyeing Kinetics

Kinetic data were obtained using a Smart Liquor equipment (Mathis^®^). During the dyeing process, the equipment measures the absorbance of the bath and generates dye depletion curves as a function of time at a rate of 6 scans per minute. These data were used to study the kinetics of the process by fitting to three kinetic models: pseudo-first order [21], pseudo-second order [22], and Weber and Morris intraparticle model (WM) [25].

## 3. Results

### 3.1. Evaluation of Surface Modification

**Figure 1:**
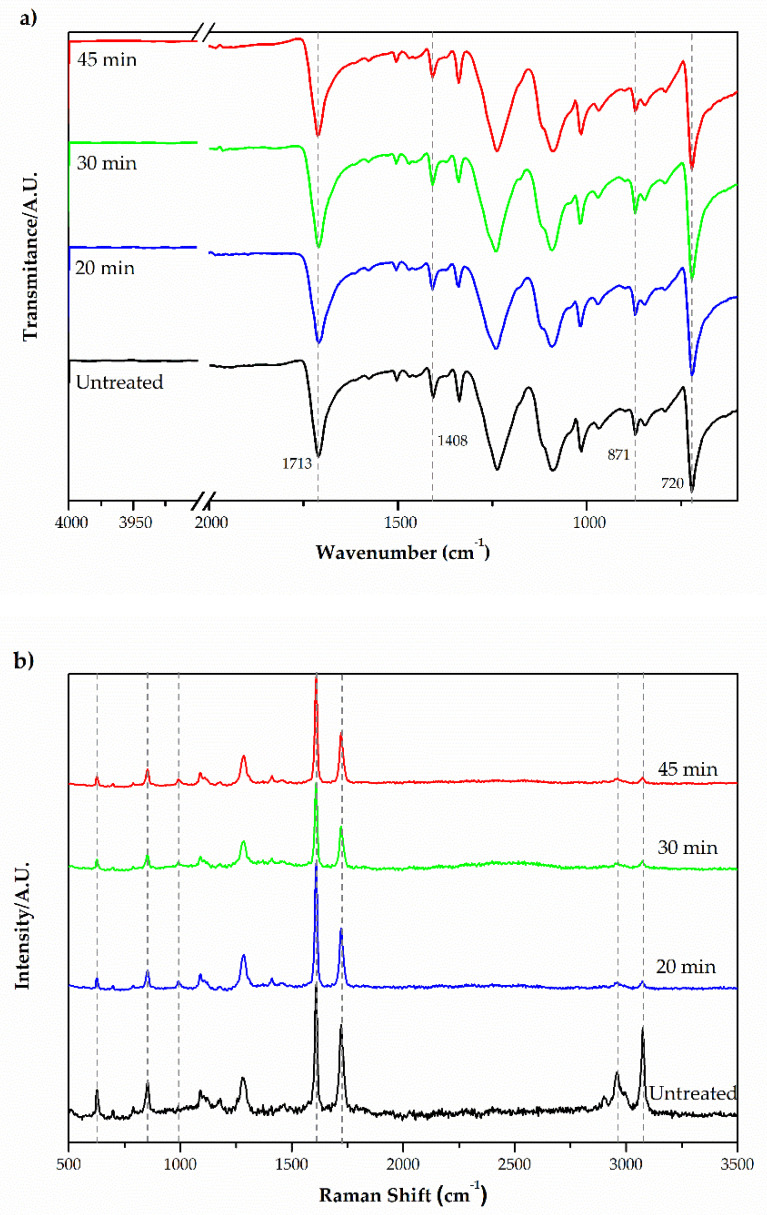
(**a**) FTIR-ATR spectra and (**b**) Raman spectra of polyester fabric samples with and without treatment at different time.

FTIR-ATR spectra of the samples is shown in Figure 1a. The characteristic bands have been identified and correlated with the chemical structure of the polyester fiber. The band at 1713 cm^−1^ refers to the carbonyl group C=O stretch, and C-O stretch and O-H deformation have been identified at 1413–1472 cm^−1^ [26,27]. The bands in the region 877 cm^−1^ are associated with the benzene ring [28]. A complementary band detected in the region 712 cm^−1^ can be attributed to the angular deformation of (CH_2_)_n_ [26].

The Raman spectra can be seen in Figure 1b. Changes in peak intensity are the result of variations in the binding energy of certain functional groups, such as those that occurred in the frequencies of 1620 cm^−1^ (C=C) and 1730 cm^−1^ (C=O). Intensity changes observed in the frequency range 500–1000 cm^−1^ correspond to variations in the vibration energy of the CH group from the aromatic rings [29]. The region between 2900 cm^−1^ and 3100 cm^−1^ shows a decrease in peaks. This region corresponds to aromatic molecules that are characteristic of the hydrophobicity of polyester [17]. The decrease in these peaks can be explained by the introduction of new functional groups such as acids and alcohols that are added via oxidation [12]. These new groups increase the polarity of the surface and enhance the interactions with the H_2_O or other polar molecules. A significant increase in intensity of peaks related to the hydrophilic bonds of the treated substrates is noted, although vibrational spectroscopy analysis do not reveal the appearance of extensive amounts of new functional groups [30]. The bands correspond to carboxylate groups at 1730 cm^−1^ and to non-aromatic functional groups at 3100 cm^−1^. It appears that the intensity of the non-aromatic bands (-CH_2_) decreases with oxidation.

Change in the surface of polyester fabric can also be probed via Zeta potential measurements. The values for Zeta potential and pH before and after a 20 min of ozone exposure can be seen in Table 1. Table 1 illustrates a significant decrease in the Zeta potential after ozone exposure. In addition, the pH referring to the zero-charge potential of the surface, also decreases upon ozone exposure. With an increase in the number of carboxylic functional groups, an increase in the negativity of the Zeta potential is expected as the dissociation of the functional groups in the water/fabric interface leads to the formation of negatively charged groups. The decrease in the pH value of the neutral surface corroborates the previous spectroscopy measurements. The increase in the number of carboxylic groups, as observed by Raman spectroscopy, leads to an increase in the possibility of deprotonation and, consequently to a decrease in the values of pH and Zeta potential.

**Figure 2:**
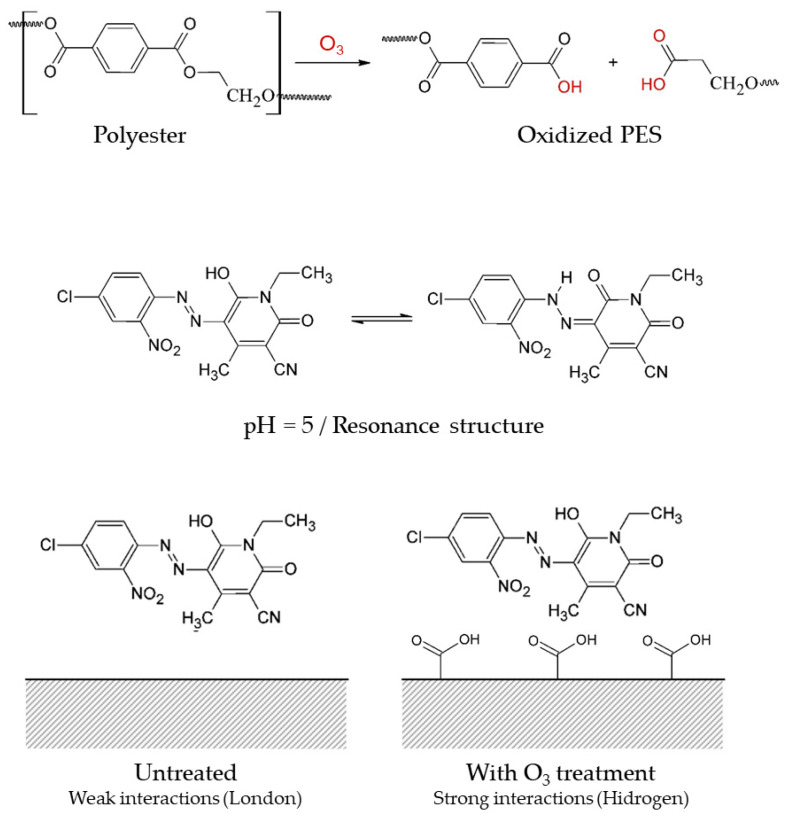
Dyeing mechanism of the C.I. Disperse Yellow 211 dye and polyester fibers.

**Figure 3:**
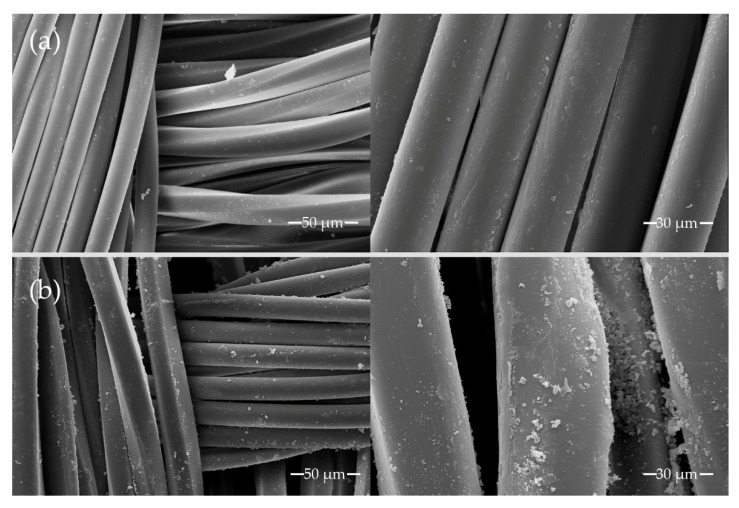
Scanning electron microscopy images of polyester fabrics after reducing bath. (**a**) Original simple, non-treated (**b**) treated with Ozone 20 min.

Figure 2 illustrates the dyeing mechanism for C.I. Disperse Yellow 211 dye and polyester fiber. The ozone modification creates highly polar ions of carboxylate and hydroxyl groups on the surface of the polyester which increase the hydrophilicity of the samples. Burkinshaw [31] showed that polyester fibers and dyes of the dispersed type have weak interactions (London type). However, with the modification of the surface by ozone, carboxylate groups offer possibilities for stronger interactions, such as hydrogen bonding between the surface -OH groups and the functional groups highlighted in red in Figure 3. It is also possible that an azo-hydrazone resonance d from the disperse dye could contribute to the formation of hydrogen bonding with ester and carboxylate groups of modified polyester, hence contributing to better interaction between dye and fabric.

These structural changes revealed by the infrared and Raman spectroscopy as well as the Zeta potential measurements may be reflected in the capillarity of the textile sample; Table 2 shows a capillarity analysis of the samples. Sample expose to ozone exhibits greater absorption as the oxidation induced by the ozone treatment appears to cause changes in both the surface and the internal structure of the polymer [32]. According to Burkinshaw [31], polyester has a low water adsorption due to the hydrophobicity of its surface groups, hence ozone treatment, which produces carboxyl and hydroxyl groups, may impart greater hydrophilicity [20]. We also observed that after a prolonged exposure of the fabric to ozone, a loss of absorption occurs. This behavior may be explained as radiation can causes degradation and erosion in the specimens [12].

### 3.2. Color Analysis

All samples had an ΔE > 1, which characterizes that none of the samples dyed after the surface modification has the same color as the standard sample. However, when analyzed using the CMC method, only sample 1 has ΔE_CMC_ > 1. The CMC method has a better colorimetric correlation between visual and tolerance assessments [33]. The sample exposed to 20 min to ozone showed a more reddish color (Δa = 2.57) and a greater color strength, of 108.65%. The data in Table 3 is in agreement with data reported by Fattahi et al. [13], who also showed that a pre-treatment with ozone increases the intensity of the color.

Table 4 shows the values of the percentage of dye reduction in the bath, in which the untreated fiber was considered as the standard condition.

According to Table 4, an increase in the time of exposure of the fabric to ozone, means a decrease in the dye to be removed from the bath. These values do illustrate the benefits of ozone treatment and are in agreement with the Rahmatinejad et al.’s [14].

The results of the color evaluation also indicate an ozone exposure of 20 min should not be exceeded in order to avoid changes in the color shade and are in agreement with the work of Eren and Anis [17]. These results confirm that the modification of the polyester structure by ozone can save energy and time, since it is possible to dye faster and to reduce the amount of dye required [10].

Figure 3 shows the morphology of the samples. The images show an agglomerated dye clusters in the fibers that received the ozone treatment (Figure 3b). The increase in the dye-fiber interaction can be related to the formation of carboxylate groups during the ozone exposure [34] which contribute to increased hydrophilicity and enable greater penetration of the dye into the fiber.

Table 5 shows the results from the friction fastness test according to ISO 105-X12 [24]. The sample exposed to 20 min of ozone, had a gray scale score of 5 for both tests, dry and wet, so, no color change was detected after the rubbing. The same behavior was noted for the sample exposed to ozone for 45 min.

**Figure 4:**
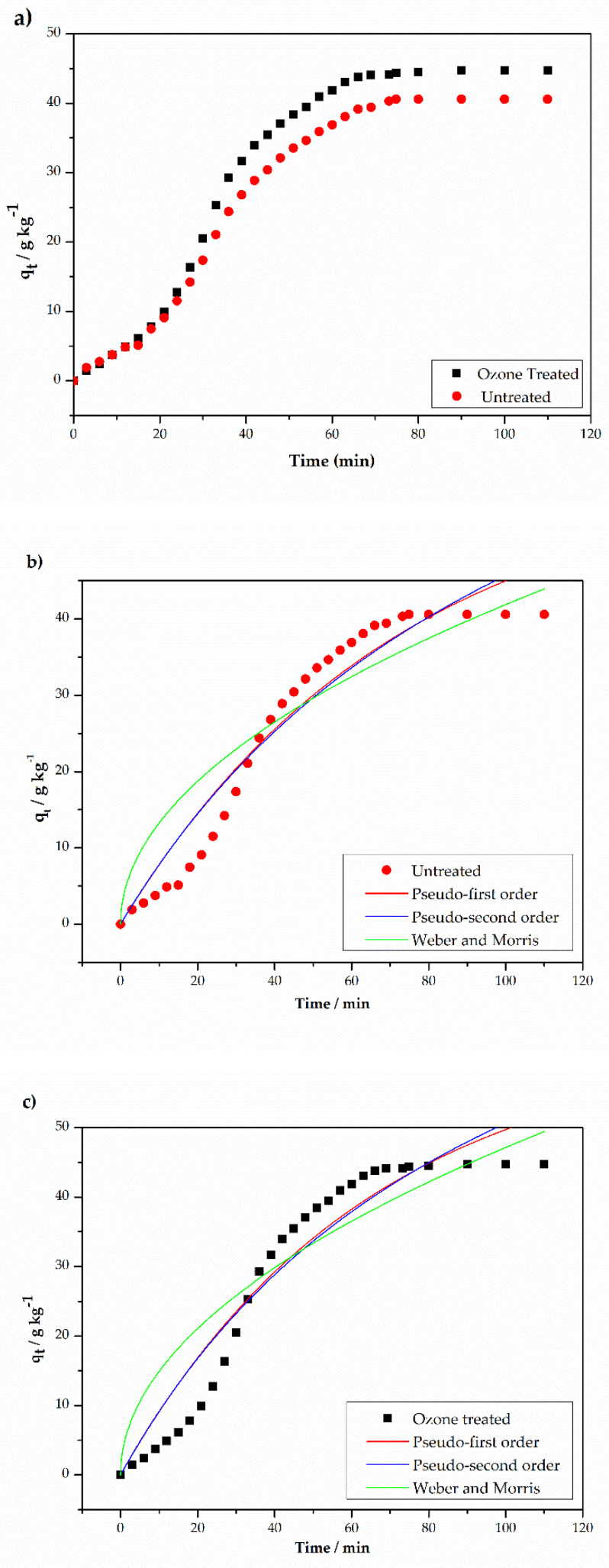
(**a**) Adsorption shape of DY211 dye in treated and untreated polyester fabric over time; (**b**) kinetic models adjustment for sample without treatment; (**c**) kinetic models adjustment for treated sample.

The time required for maximum dye adsorption is called equilibrium time, after which the adsorption remains constant. Figure 4a shows the adsorption times for two samples of fabric, one being treated with ozone and one without treatment. Figure 4a shows an equilibrium time of 72 min for both samples. The kinetic data for the dyeing processes was analyzed according to the kinetic models of pseudo-first order, pseudo-second order, and the Weber and Morris intraparticle diffusion models. The fitting results are shown in Figure 4.

Table 6 shows the parameters of the kinetic models obtained by adjusting the models to the experimental data for the untreated and treated samples. None of the adopted models showed good agreement, indicating that the dye adsorption process may be taking place in more than one stage. In the case of polyester, the interaction of the dispersed dye with the fiber occurs as a function of temperature, since its increase causes the fibers to swell, allowing the dye to be inserted [35]. As swelling is not uniform, and the initial interaction between dye and fiber is greater on the surface layer, it can be understood that there is more than one diffusion process. These processes occur until the formation of the horizontal asymptote, with q_e_ = 40.23 g·kg^−1^ and 44.55 g·kg^−1^ for treated and untreated material, an increase in adsorption of 10.74%. These results are similar to those reported by Lee et al. [32], which indicate an increase in dye adsorption as a function of ozone exposure.

**Figure 5:**
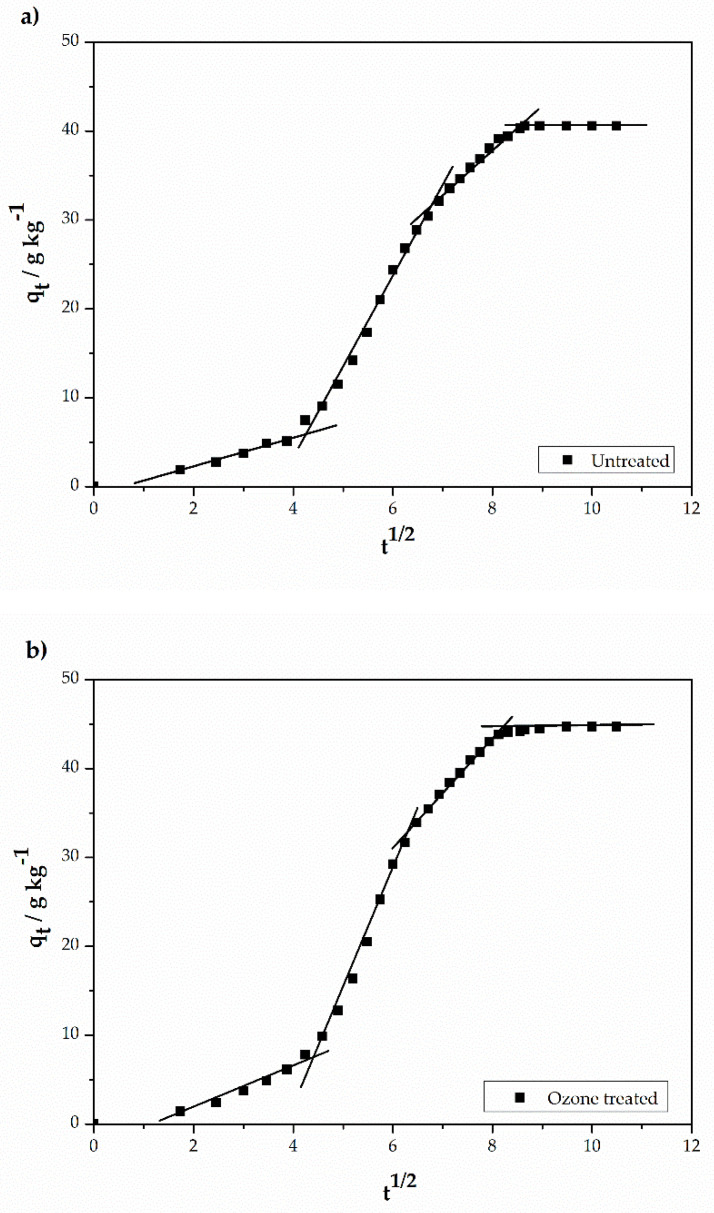
Intra-particle kinetic diffusion for dye adsorption in (**a**) untreated and (**b**) ozone-treated polyester.

According to the Weber and Morris model, if the plot q_t_ vs. t^1/2^ is linear and passes through the origin, intra-particle diffusion is the rate-limiting step. However, multi-linearities are observed in Figure 5, indicating that at least three adsorption steps occur during the dyeing processes with different intraparticle diffusion rates. Each of the linear sections was evaluated separately and the parameters obtained are listed in Table 7.

The first portion of the plot in Figure 5 is generally attributed to the diffusion of the dye through the diffusional boundary layer that exists at the fiber’ surface. In this section we observe that the lowest diffusion rate is due to the existence of a major effective resistance to the dye particle’s diffusion. The linear portion of the first step does not pass through the origin indicating that intraparticle transport is not the only rate-limiting step and that there is some degree of boundary layer control [35].

The second portion describes the adsorption of the dye onto sites localized on the fiber surface [36] and has the highest diffusion rate due to a smaller resistance to reach adsorption sites. That is the case for both treated and untread samples. This process takes place fast until it reaches saturation of the fiber surface, followed by diffusion of the dye molecules inside the fiber (the third portion). The third section illustrates a process that follows a slower rate due to the presence of fewer adsorption sites available.

## 4. Conclusions

We report on the modification of polyester fabrics with ozone and the dyeing of the modified fabrics with the C.I. Disperse Yellow 211 dye. We observed molecular and topographical changes in the polyester fabric after ozone exposure. While FT-IR and Raman spectra do not reveal the appearance of extensive amounts of new functional groups on the treated fabrics, Raman spectra shows that the intensity of the non-aromatic bands decreases with oxidation and oxidized groups are created by ozonolysis, with a significant increase in the intensity of peaks related to the hydrophilic bonds. Friction fastness tests shows that upon 20 min of ozone exposure, the specimens have a score of 5 for both tests, dry and wet. Kinetic data show that dye adsorption process can be explained by a three-steps Weber and Morris intraparcicle diffusion model. The amount of dye adsorption at equilibrium is higher by a 10.74% for the untreated samples confirming that ozone treatment may be an efficient means to reduce the amount of dye used. We assume that this increase is due to an increase in hydrogen bond interactions after ozone treatment. To emphasize the nature of structural changes and the different possibilities of interaction between dye and fabric, we will use aromatic dyes, without hydrogen bond probability, for future work to improve the understanding of this process. This work shows that ozone treatment is a good way to reduce inputs in the dyeing processes and also wastewater treatment expenses, thus, reducing the environmental impacts of the textile industry as well as potentially decreasing the processing costs.

## Figures and Tables

**Table 1:** Results of Zeta potential of polyester samples before and after 20 min of ozone treatment.

Sample	Side	Potential Zeta/mV	pH
Untreated	A	−3.92 ± 0.08	6.14 ± 0.03
	B	−3.65 ± 0.02	6.15 ± 0.03
Ozone 20 min treated	A	−8.22 ± 0.26	6.04 ± 0.03
	B	−7.46 ± 0.15	6.05 ± 0.03

**Table 2:** Capillarity tests for polyester fabrics as a function of ozone treatment at different exposure times.

Sample	Absorption Height (cm)
Untreated	0
Ozone treated 20 min	2
Ozone treated 30 min	1
Ozone treated 45 min	0.5

**Table 3:** Color assessment of ozone-treated polyester fabrics compared to untreated fabric.

Parameters	Ozone Treated (min)		
	20	30	40
ΔL	0.04	0.89	0.96
Δa	2.57	0.96	1.00
Δb	1.44	2.13	1.99
ΔC	1.19	2.02	1.87
ΔH	−2.70	−1.17	−1.20
ΔE	2.95	2.52	2.40
ΔE_CMC_	1.36	0.90	0.87
*Strength (%)*	108.65	104.08	103.58

**Table 4:** Correction of the dyeing bath in % of dye to be removed.

Ozone Treated Sample (min)	% Dye to Be Removed from the Bath
20	−7.96%
30	−3.92%
45	−3.46%

**Table 5:** Polyester fabric friction fastness score of samples.

Sample	Score Dry	Score Wet
Untreated	4	4
Ozone treated 20 min	5	5
Ozone treated 30 min	4	5
Ozone treated 45 min	4	4

**Table 6:** Kinetic parameters for the non-linear models of adsorption.

Sample	Parameter	PFO	PSO	WM
Untreated	q_e_	59.51493 ± 7.20762	98.38993 ± 17.53605	-
	k_1_	0.01403 ± 0.00274	-	-
	k_2_	-	0.00009 ± 0.00004	-
	K_WM_	-	-	4.18392 ± 0.15833
	R^2^	0.93991	0.93357	0.84508
Ozone treated 20 min	q_e_	62.80174 ± 7.535222	101.90946 ± 18.4794	-
	k_1_	0.00316 ± 0.00316	-	-
	k_2_	-	0.00010 ± 0.00005	-
	K_WM_	-	-	4.69444 ± 0.19026
	R^2^	0.92747	0.91937	0.83371

PFO = pseudo-first order model; PSO = pseudo-second order model; WM = Weber and Morris intraparticle diffusion model.

**Table 7:** Parameters of the intraparticle kinetic models together with its regression coefficients of adsorption data sample without treatment and 20′ ozone treatment.

Sample	Parameter	
Untreated	K_1_ (mg g^−1^ min^−0.5^)	1.6041 ± 0.1315
	R^2^	0.9751
	K_2_ (mg g^−1^ min^−0.5^)	9.9810 ± 0.3385
	R^2^	0.9886
	K_3_ (mg g^−1^ min^−0.5^)	5.1431 ± 0.2383
	R^2^	0.9831
Ozone treated 20 min	K_1_ (mg g^−1^ min^−0.5^)	2.5028 ± 0.2429
	R^2^	0.9546
	K_2_ (mg g^−1^ min^−0.5^)	13.8194 ± 0.5721
	R^2^	0.9898
	K_3_ (mg g^−1^ min^−0.5^)	6.0526 ± 0.1408
	R^2^	0.9957

## Data Availability

Not applicable.

## References

[1] Navaneetha Pandiyaraj K., Vasu D., Ramkumar M.C., Deshmukh R.R., Ghobeira R. (2021). Improved Degradation of Textile Effluents via the Synergetic Effects of Cu-CeO_2_ Catalysis and Non-Thermal Atmospheric Pressure Plasma Treatment. Sep. Purif. Technol..

[2] Khan S., Anas M., Malik A. (2019). Mutagenicity and Genotoxicity Evaluation of Textile Industry Wastewater Using Bacterial and Plant Bioassays. Toxicol. Rep..

[3] Samuchiwal S., Bhattacharya A., Malik A. (2020). Treatment of Textile Effluent Using an Anaerobic Reactor Integrated with Activated Carbon and Ultrafiltration Unit (AN-ACF-UF Process) Targeting Salt Recovery and Its Reusability Potential in the Pad-Batch Process. J. Water Process Eng..

[4] Chequer F.M.D., Dorta D.J., Oliveira D.P. (2011). de Azo Dyes and Their Metabolites: Does the Discharge of the Azo Dye into Water Bodies Represent Human and Ecological Risks?. Adv. Treat. Text. Effl..

[5] Carneiro P.A., Umbuzeiro G.A., Oliveira D.P., Zanoni M.V.B. (2010). Assessment of Water Contamination Caused by a Mutagenic Textile Effluent/Dyehouse Effluent Bearing Disperse Dyes. J. Hazard. Mater..

[6] Pang Y.L., Abdullah A.Z. (2013). Current Status of Textile Industry Wastewater Management and Research Progress in Malaysia: A Review. Clean Soilairwater.

[7] Lade H.S., Waghmode T.R., Kadam A.A., Govindwar S.P. (2012). Enhanced Biodegradation and Detoxification of Disperse Azo Dye Rubine GFL and Textile Industry Effluent by Defined Fungal-Bacterial Consortium. Int. Biodeterior. Biodegrad..

[8] De Souza O., de Oliveira L.J. (2016). GlobalizaçãO e relações de consumo: ServidãO moderna e degradação ambiental. Rev. Direito Ambient. Soc..

[9] Burkinshaw S.M., Liu K., Salihu G. (2019). The Wash-off of Dyeings Using Interstitial Water Part 5: Residual Dyebath and Wash-off Liquor Generated during the Application of Disperse Dyes and Reactive Dyes to Polyester/Cotton Fabric. Dye. Pigment..

[10] Eren H.A. (2006). Afterclearing by Ozonation: A Novel Approach for Disperse Dyeing of Polyester. Coloration Technol..

[11] Choi H., Kim Y.Y., Lim H., Cho J., Kang J.W., Kim K.S. (2001). Oxidation of Polycyclic Aromatic Hydrocarbons by Ozone in the Presence of Sand. Water Sci. Technol..

[12] Jang J., Jeong Y. (2006). Nano Roughening of PET and PTT Fabrics via Continuous UV/O3 Irradiation. Dye. Pigment..

[13] Fattahi F., Izadan H., Khodami A. (2012). Investigation into the Effect of UV/Ozone Irradiation on the Dyeing behaviour of Poly(Lactic Acid) and Poly(Ethylene Terephthalate) Substrates. Prog. Colorcolorants Coat..

[14] Rahmatinejad J., Khoddami A., Mazrouei-Sebdani Z., Avinc O. (2016). Polyester Hydrophobicity Enhancement via UV-Ozone Irradiation, Chemical Pre-Treatment and Fluorocarbon Finishing Combination. Prog. Org. Coat..

[15] Atav R., Yurdakul A. (2011). Effect of the Ozonation Process on the Dyeability of Mohair Fibres. Coloration Technol..

[16] Atav R., Namirti O. (2016). Effect of Ozonation Process on Dyeing of Polyamide Fabrics with a Natural Dye: Walnut Rind/Efectul Procesului de Ozonizare Asupra Vopsirii Tesaturilor de Poliamida Cu Colorant Natural Din Coaja de Nuca. Ind. Text..

[17] Eren H.A., Anis P. (2009). Surface Trimer Removal of Polyester Fibers by Ozone Treatment. Text. Res. J..

[18] Wakida T., Lee M., Jeon J.H., Tokuyama T., Kuriyama H., Ishida S. (2004). Ozone-Gas Treatment of Wool and Silk Fabrics. J. Fiber Sci. Technol..

[19] He Z., Li M., Zuo D., Xu J., Yi C. (2019). Effects of Color Fading Ozonation on the Color Yield of Reactive-Dyed Cotton. Dye. Pigment..

[20] Dos Santos V.L.V.F., Barcellos I.O., Piccoli H.H., dos Santos V.L.V.F., Barcellos I.O., Piccoli H.H. (2017). Pre-Alvejamento de Materiais Têxteis Com Ozônio e Avaliação de Suas Propriedades de Superfície, Físicas e Tintoriais. Matéria.

[21] Lagergren S. (1898). Zur theorie der sogenannten adsorption gelöster stoffe, kungliga svenska vetenskapsakademiens. Handlingar.

[22] Ho Y.S., McKay G. (1999). Pseudo-Second Order Model for Sorption Processes. Process Biochem..

[23] Largitte L., Pasquier R. (2016). A Review of the Kinetics Adsorption Models and Their Application to the Adsorption of Lead by an Activated Carbon. Chem. Eng. Res. Des..

[24] (2016). Test for Colour Fastness—Part X12: Colour Fastness to Rubbing.

[25] Plazinski W., Dziuba J., Rudzinski W. (2013). Modeling of Sorption Kinetics: The Pseudo-Second Order Equation and the Sorbate Intraparticle Diffusivity. Adsorption.

[26] Fechine G.J.M., Rabello M.S., Souto-Maior R.M. (2002). The Effect of Ultraviolet Stabilizers on the Photodegradation of Poly(Ethylene Terephthalate). Polym. Degrad. Stab..

[27] Edge M., Wiles R., Allen N.S., McDonald W.A., Mortlock S.V. (1996). Characterisation of the Species Responsible for Yellowing in Melt Degraded Aromatic Polyesters—I: Yellowing of Poly(Ethylene Terephthalate). Polym. Degrad. Stab..

[28] Iyer P.B., Iyer K.R.K., Patil N.B. (1976). An Infrared Technique for the Quick Analysis of Cotton–Polyester Blends. J. Appl. Polym. Sci..

[29] Costa T.H.C., Feitor M.C., Alves Junior C., Bezerra C.M. (2008). Caracterização de Filmes de Poliéster Modificados Por Plasma de O_2_ a Baixa Pressão. Matéria.

[30] Van Geluwe S., Vinckier C., Braeken L., Van der Bruggen B. (2011). Ozone Oxidation of Nanofiltration Concentrates Alleviates Membrane Fouling in Drinking Water Industry. J. Membr. Sci..

[31] Burkinshaw S.M. (2016). Polyester Fibres. Physico-Chemical Aspects of Textile Coloration.

[32] Lee M., Lee M.S., Wakida T., Tokuyama T., Inoue G., Ishida S., Itazu T., Miyaji Y. (2006). Chemical Modification of Nylon 6 and Polyester Fabrics by Ozone-Gas Treatment. J. Appl. Polym. Sci..

[33] Ruyter I.E., Nilner K., Moller B. (1987). Color Stability of Dental Composite Resin Materials for Crown and Bridge Veneers. Dent. Mater..

[34] Gupta B., Hilborn J., Hollenstein C., Plummer C.J.G., Houriet R., Xanthopoulos N. (2000). Surface Modification of Polyester Films by RF Plasma. J. Appl. Polym. Sci..

[35] Özcan A.S., Özcan A. (2005). Adsorption Behavior of a Disperse Dye on Polyester in Supercritical Carbon Dioxide. J. Supercrit. Fluids.

[36] Lis M.J., Valldeperas J., Carrillo F. (2006). Análisis cinético y matemático de la tintura de tencel con colorantes directos. Boletín Intexter.

